# Analysis of the Urine

**Published:** 1886-12

**Authors:** 


ANALYSIS OF THE URINE. By K. B. Hofmann, Professor in the University of Gratz, and R. Ultzmann, Docent in the University of Vienna. Translated by T. Barton Brune, A. M., M. D., and H. Holbrook Curtis, Ph. B., M. D. Second edition. D. Appleton & Co., New York. 1886. 8vo., pp. 310.

There seems to be a growing demand for practical literary work, and writers are beginning to realize the fact by condensing the data of a subject as far as possible and bringing out the facts in clear relief. The work before us is a decided success in this particular, and for a concise, yet complete and thoroughly reliable exposition of urinary analysis, it stands unique.

The reputation of its authors is a sufficient guarantee for its trustworthiness. The scientific practitioner of to-day recognizes the importance of the chemical and microscopical examination of the urine in the diagnosis of genito-urinary disorders, especially renal disease, and it is one of the leading features of the work to give the characteristic, diagnostic points of each diseased process, together with a clear description of the changes in the urine. Chapter VII, General Diagnosis, and chapter VIII, Diagnosis of Diseases of the Urinary Apparatus, are devoted to this matter, and are invaluable as time-saving guides. At the end of the book are eight colored plates, showing the nature of various urine sediments. The mechanical execution of the book is superb, and in every way sustains the excellent reputation of the publishers.

